# Supplementary material for: High prevalence of intestinal schistosomiasis in school-age children in the villages adjacent to Lake Chamo in the southern Rift Valley of Ethiopia
Source: PLoS Negl Trop Dis. 2024 Oct 29;18(10):e0012613. doi: 10.1371/journal.pntd.0012613 (PMC11548786; doi:10.1371/journal.pntd.0012613)
Supplement: Attachment — Submitted filename: PNTD-D-24-00946_reviewer.pdf [file pntd.0012613.s001.pdf]

PLOS Neglected Tropical Diseases

High prevalence of intestinal schistosomiasis in school-age children in the villages adjacent to Lake Chamo in southern Rift Valley of Ethiopia: an implication for schistosomiasis deworming?

--Manuscript Draft--

**Manuscript Number:**	PNTD-D-24-00946
**Full Title:**	High prevalence of intestinal schistosomiasis in school-age children in the villages adjacent to Lake Chamo in southern Rift Valley of Ethiopia: an implication for schistosomiasis deworming?
**Short Title:**	Schistosoma mansoni
**Article Type:**	Research Article
**Keywords:**	Schistosoma mansoni; school age children; Shelle Mela; South Ethiopia
**Abstract:**	Background<br><br>The prevalence of intestinal schistosomiasis remains a challenge despite government efforts to eliminate the disease. This study aims to assess the prevalence of intestinal schistosomiasis in school-age children living in the villages surrounding Lake Chamo in southern Rift Valley of Ethiopia.<br><br>Methodology/Principal Findings<br><br>A cross-sectional study was conducted from January to July 2023 in Shelle Mela Kebele in Gamo Zone, South Ethiopia. Stool samples were collected from 597 school-age children. A singe Kato-Katz for helminths and formalin-ether concentration technique for protozoan parasites were used to process the samples. The intensity of Schistosoma mansoni infection among school-age children was determined by counting the number of eggs per gram of stool. Of the 597 children screened, 52.3% (95% CI: 48.4.4–56.4) were positive for Schistosoma mansoni. These findings showed that 55% of the infections were light, 30.3% were moderate, and 14.7% were heavy. The mean egg count of S. mansoni parasites was 182.1 eggs per gram. The prevalence of other intestinal parasites (Hymenolopis nana, Ascaris lumbricoides, Hookworm, Taenia species, Giardia lamblia and Entamoeba histolytica) was found to be 7.7% (46/597). The prevalence of S. mansoni co-infection with all other intestinal parasites was 5.0% (30/597). The co-infection with A. lumbricoides was observed in 9 cases (1.5%), with H. nana in 8 cases (1.3%), with Taenia species in 6 cases (1.0%), with Hookworm in 1 case (0.2%), with E. histolytica in 1 case (0.2%), and with G. lamblia in 1 case (0.2%).<br><br>Conclusions/significances<br><br>The study showed a high rate of S. mansoni infection among school-age children. This calls for immediate action, such as school-based deworming, to protect these children from the disease and reduce the burden. Further research is needed to understand the transmission of the infection by the intermediate host.
**Additional Information:**	
**Question**	**Response**
**Government Employee**<br><br>Are you or any of the contributing authors an employee of the United States government?	No - No authors are employees of the U.S. government.

Powered by Editorial Manager® and ProduXion Manager® from Aries Systems Corporation

Manuscripts authored by one or more US Government employees are not copyrighted, but are licensed under a CC0 Public Domain Dedication, which allows unlimited distribution and reuse of the article for any lawful purpose. This is a legal requirement for US Government employees.<br><br>This will be typeset if the manuscript is accepted for publication.	
**Financial Disclosure**<br><br>Enter a financial disclosure statement that describes the sources of funding for the work included in this submission. Review the submission guidelines for detailed requirements. View published research articles from *PLOS Neglected Tropical Diseases* for specific examples.<br><br>This statement is required for submission and **will appear in the published article** if the submission is accepted. Please make sure it is accurate.<br><br>**Funded studies**<br>Enter a statement with the following details:<br>• Initials of the authors who received each award<br>• Grant numbers awarded to each author<br>• The full name of each funder<br>• URL of each funder website<br>• Did the sponsors or funders play any role in the study design, data collection and analysis, decision to publish, or preparation of the manuscript?<br><br>Did you receive funding for this work?	Yes
Please add funding details.<br>as follow-up to "**Financial Disclosure**<br><br>Enter a financial disclosure statement that describes the sources of funding for the work included in this submission. Review the submission guidelines for detailed requirements. View published research	The Norwegian Programme for Capacity Development in Higher Education and Research for Development (QZA-21/0162)

articles from *PLOS Neglected Tropical Diseases* for specific examples.<br><br>This statement is required for submission and **will appear in the published article** if the submission is accepted. Please make sure it is accurate.<br><br>**Funded studies**<br>Enter a statement with the following details:<br>• Initials of the authors who received each award<br>• Grant numbers awarded to each author<br>• The full name of each funder<br>• URL of each funder website<br>• Did the sponsors or funders play any role in the study design, data collection and analysis, decision to publish, or preparation of the manuscript?<br><br>Did you receive funding for this work?"	
Please select the country of your main research funder (please select carefully as in some cases this is used in fee calculation).<br>as follow-up to "**Financial Disclosure**<br><br>Enter a financial disclosure statement that describes the sources of funding for the work included in this submission. Review the submission guidelines for detailed requirements. View published research articles from *PLOS Neglected Tropical Diseases* for specific examples.<br><br>This statement is required for submission and **will appear in the published article** if the submission is accepted. Please make sure it is accurate.	NORWAY - NO

Powered by Editorial Manager® and ProduXion Manager® from Aries Systems Corporation

**Funded studies**<br>Enter a statement with the following details:<br>• Initials of the authors who received each award<br>• Grant numbers awarded to each author<br>• The full name of each funder<br>• URL of each funder website<br>• Did the sponsors or funders play any role in the study design, data collection and analysis, decision to publish, or preparation of the manuscript?<br><br>Did you receive funding for this work?"	
**Competing Interests**<br><br>On behalf of all authors, disclose any competing interests that could be perceived to bias this work.<br><br>This statement will be typeset if the manuscript is accepted for publication.<br><br>Review the instructions link below and PLOS NTDs' competing interests policy to determine what information must be disclosed at submission.	All authors have declared that they do not have any competing interests
**Data Availability**<br><br>Provide a **Data Availability Statement** in the box below. This statement should detail where the data used in this submission can be accessed. This statement will be typeset if the manuscript is accepted for publication.<br><br>Before publication, authors are required to make all data underlying their findings fully available, without restriction. Review our PLOS Data Policy page for detailed information on this policy. Instructions for writing your Data Availability statement can be accessed via the Instructions link below.	All relevant data are within the manuscript.

Manuscript Click here to access/download;Manuscript;Manuscript.docx

High prevalence of intestinal schistosomiasis in school-age children in the villages adjacent to Lake Chamo in southern Rift Valley of Ethiopia: an implication for schistosomiasis deworming?

Yenenesh Ayele[1], Teklu Wegayehu[1], Daniel Woldeyes[1], Fekadu Massebo[1]*

[1]Department of Biology, Arba Minch University, Arba Minch, Ethiopia

*Corresponding author: fekadu.massebo@amu.edu.et; fekadu_massebo@yahoo.com (FM)

Abstract

Background

The prevalence of intestinal schistosomiasis remains a challenge despite government efforts to eliminate the disease. This study aims to assess the prevalence of intestinal schistosomiasis in school-age children living in the villages surrounding Lake Chamo in southern Rift Valley of Ethiopia.

Methodology/Principal Findings

A cross-sectional study was conducted from January to July 2023 in Shelle Mela *Kebele* in Gamo Zone, South Ethiopia. Stool samples were collected from 597 school-age children. A singe Kato-Katz for helminths and formalin-ether concentration technique for protozoan parasites were used to process the samples. The intensity of *Schistosoma mansoni* infection among school-age children was determined by counting the number of eggs per gram of stool. Of the 597 children screened, 52.3% (95% CI: 48.4.4–56.4) were positive for *Schistosoma mansoni*. These findings showed that 55% of the infections were light, 30.3% were moderate, and 14.7% were heavy. The mean egg count of *S. mansoni* parasites was 182.1 eggs per gram. The prevalence of other intestinal parasites (*Hymenolopis nana, Ascaris lumbricoides*, Hookworm, *Taenia* species, *Giardia*

lamblia and *Entamoeba histolytica*) was found to be 7.7% (46/597). The prevalence of *S. mansoni* co-infection with all other intestinal parasites was 5.0% (30/597). The co-infection with *A. lumbricoides* was observed in 9 cases (1.5%), with *H. nana* in 8 cases (1.3%), with *Taenia* species in 6 cases (1.0%), with Hookworm in 1 case (0.2%), with *E. histolytica* in 1 case (0.2%), and with *G. lamblia* in 1 case (0.2%).

Conclusions/significances

The study showed a high rate of *S. mansoni* infection among school-age children. This calls for immediate action, such as school-based deworming, to protect these children from the disease and reduce the burden. Further research is needed to understand the transmission of the infection by the intermediate host.

Keywords: *Schistosoma mansoni*, school age children, Shelle Mela, South Ethiopia

Author summary

Schistosomiasis is a neglected tropical parasitic disease that affects over 220 million people globally. Although there is a deworming mass drug administration (MDA) program for soil-transmitted helminths, the study area still needs to be integrated into the Praziquantel MDA. The study focused on school-age children to better understand the prevalence of the disease and recommend interventions. Hence, we assessed the prevalence and intensity of intestinal schistosomiasis among school-age children living in selected villages near Lake Chamo in the southern Rift Valley of Ethiopia. The results revealed that the prevalence of *Schistosoma mansoni* was 52.3%. The overall prevalence of other intestinal parasites (*Hymenolopis nana*, *Ascaris lumbricoides*, Hookworm, *Taenia* species, *Giardia lamblia*, and *Entamoeba histolytica*) was 7.7%. We documented a 5% *S. mansoni* co-infection with all other intestinal parasites including *A. lumbricoides*, *H. nana, Taenia* species, Hookworm, *E. histolytica*, and *G. lamblia*. This implies a high prevalence of intestinal parasites in school-age children, necessitating immediate action to alleviate this burden. Implementing school-based mass drug administration (MDA) for schistosomiasis is advisable in the study villages.

Introduction

Schistosomiasis is a neglected tropical parasitic disease that affects more than 220 million people worldwide [1]. The tropical and sub-tropical regions are known to harbor the poorest individuals mostly affected by this disease [2]. Sub-Saharan African countries bear more than 90% of the schistosomiasis burden [3].

Seven species of *Schistosoma* parasites infect humans, namely *Schistosoma mansoni, Schistosoma haematobium, Schistosoma japonicum, Schistosoma mekongi, Schistosoma intercalatum, Schistosoma guineensis*, and *Schistosoma malayensis* [4]. *Schistosoma*

mansoni, S. haematobium and *S. japonicum* are the most commonly found in human infections, with *S. mansoni* and *S. haematobium* being more prevalent in Africa [5]. Many parts of Africa have snail species that act as intermediate hosts for schistosomiasis. *Biomphalaria* species are intermediate hosts of intestinal schistosomiasis due to *Schistosoma mansoni,* while *Bulinus* species are intermediate hosts of urogenital schistosomiasis [6]. Schistosomiasis is a prevalent disease in Ethiopia, putting millions of people at risk of infection [7]. Two types of parasites, *Schistosoma mansoni* and *S. haematobium*, are commonly found in many areas in Ethiopia [8].

The primary strategy for combating schistosomiasis involves mass deworming individuals at risk using Praziquantel [1]. Ethiopia adheres to the WHO guidelines and implements mass drug administration (MDA) for school-age children as part of its deworming program whenever eligible for the MDA. While this approach has effectively reduced the disease burden in the community, it does not prevent reinfection if individuals come into contact with contaminated water sources [1]. Therefore, it is necessary to manage snails and maintain a hygienic environment to prevent water pollution from human waste.

In villages near Ethiopia's southern Rift Valley lakes, a recent study revealed that most people rely on Lake Chamo and rivers for irrigation, fishing, and other household activities. This increased contact with water bodies puts them at a higher risk of *Schistosoma* infection [9]. Despite the government's efforts to eliminate the disease, health centers in the study area continue to report cases of schistosomiasis. Apart from the soil transmitted helminthes deworming programme, the study area has not been integrated into the Praziquantel MDA. The study focused on school-age children to better understand the prevalence of the disease and recommend interventions. The findings support implementing necessary preventive measures for those at higher risk of infection. The data could help in disease prevention efforts at healthcare facilities and

various governmental and non-governmental organizations. The study aimed to determine the prevalence and intensity of intestinal schistosomiasis among school-age children living in selected villages near Lake Chamo in Ethiopia's southern Rift Valley lakes.

Materials and methods

Study area description

The study was conducted in Shelle Mela Kebele, in the Arba Minch area district of southwest Ethiopia (Figure 1). The Kebele consists of five sub-villages near Lake Chamo. Two health posts offer primary health care services in conjunction with the Kolla Shelle Health Centre, located about 35 kilometers from Arba Minch town, the administrative center for the Gamo Zone.

Most people in the study area depend on agriculture as their main source of income. They cultivate crops like bananas, avocados, mangoes, and maize and rely on irrigation from Lake Chamo, the Sille, and Sago rivers, which flow into the lake. It's important to note that intestinal schistosomiasis has been recorded in the health centre registration book and is a significant public health concern in the region. The availability of water body and high contact of people with water for washing clothes, irrigate their plantation and fishing could increase the risk of the schistosomiasis. To address this issue, the government has launched a deworming program focusing on school to combat soil-transmitted helminthes.

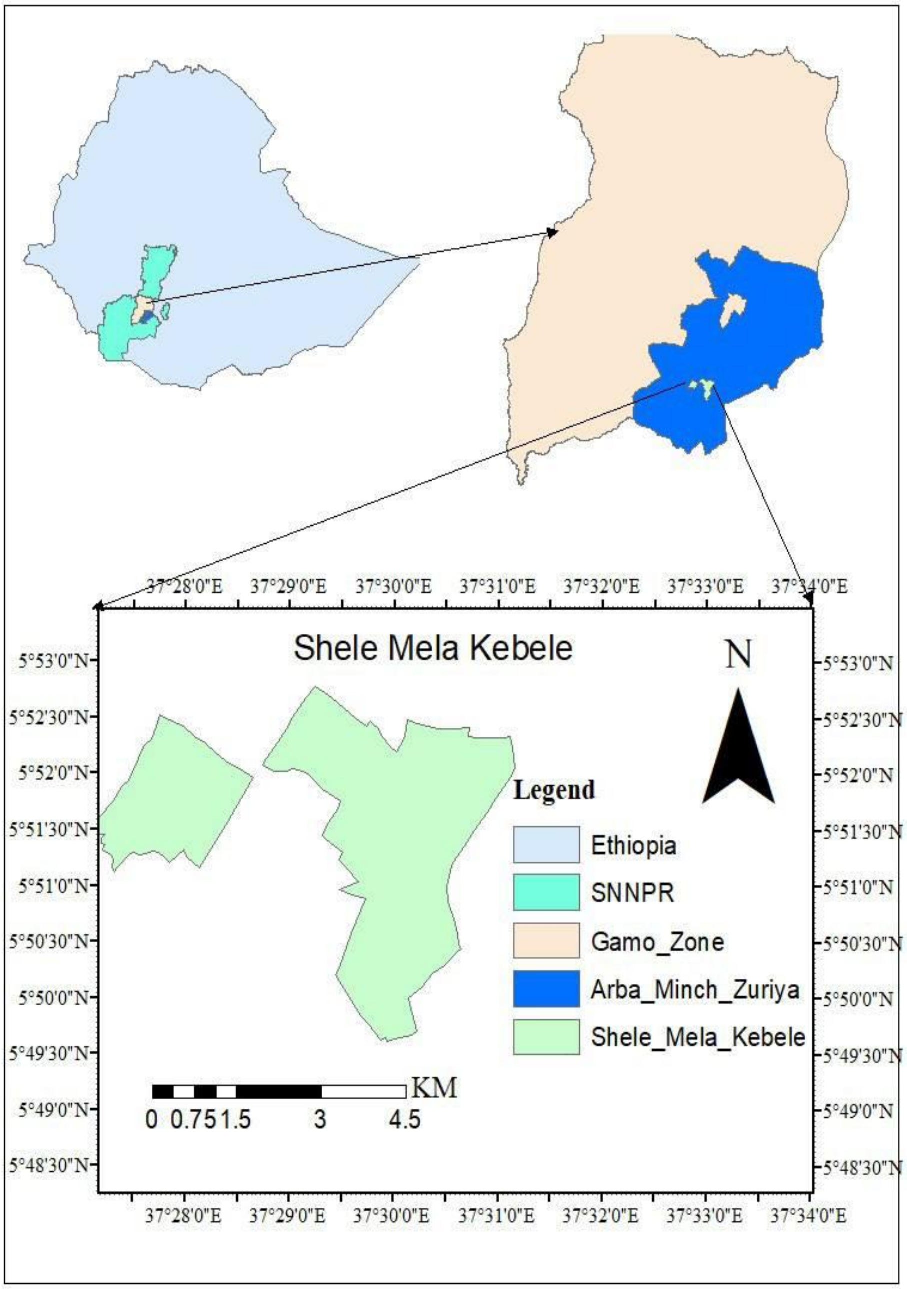

Figure 1. Map of Shelle Mela *Kebele* Arba Minch *Zuria* district, Gamo zone, south Ethiopia

Study design and period

A community-based cross-sectional study was conducted from January to July 2023 to determine the prevalence of intestinal schistosomiasis and other intestinal parasitic infections in school-age children.

Study population, study participants and sampling technique

The study population was all school-age children (age 5 to 15) in Shelle Mela *Kebele*. We randomly selected households with school-age children in each sub-village to ensure our samples were representative. Trained data collectors visited the selected households, explained the study's purpose, and obtained informed consent from the parents or guardians of the children. The children were then given instructions on how to collect and provide stool samples for analysis. We also prepared a questionnaire in English, which we translated into the local language (Gammogna) to gather socio-demographic information about the study participants.

Sample size determination

To get the sample size, the single population proportion formula was used. This study comprised 597 school-age children. Because there had been no previous research in the area, the sample size for the study was calculated using 50% of the prevalence.

Therefore, the sample was calculated as:

$$n = \frac{(Z\,\alpha/2)2 * \;Pq}{(d)\;2}$$

Where, Z= ~~is table~~ value of 5% level of significance (z = 1.96)

α = is level of significance (α = 0.05 = 5%),

d= is error tolerance (d = 0.04),

p= proportion of success (p = 0.5 = 50%)

q= proportion of failure (q =1-p) = 0.5

Inclusion and exclusion criteria

The study enrolled all school-aged children who lived in the selected households and voluntarily provided stool samples. Any child who was ill and receiving medication was excluded from the study. In addition, children whose parents or guardians did not provide consent were also excluded. Also, children who did not voluntarily provide stool samples were excluded.

Stool sample collection and examination

The children were informed how to collect stool samples without contamination. They were provided applicator sticks, clean paper, and a labelled stool cup with details about the participant, like age and sex. Some portion of individual stool sample was taken for Kato-Katz thick smear preparation in the field. All the rest portion of collected samples were placed in an ice box container and transported to the parasitological laboratory at the College of Medicine and Health Sciences for diagnosis on the same day.

Kato-Katz technique

Stool samples were collected from children in selected households, and a smear was prepared according to the manufacturer's instructions for Kato-Katz thick smear preparation. An applicator stick was used to transfer a small portion of the stool sample onto a mesh-covered paper, which was then strained and screened with a spatula. The filtered sample was placed in the centre of a glass slide, and a wallboard was used to fill

the hole in the centre with the sample. After removing the wallboard, the glass slide was covered with malachite green-soaked cellophane tape and pressed with another clean slide to spread the sample throughout cellophane tape cover.

The slides were examined under an Olympus CX31 microscope with low-objective lenses (10 xs or 40 xs) within an hour of smear preparation. Positive results were confirmed with a medium power objective lens. The number of helminths eggs present in the whole smear was counted and documented on the laboratory report form designed for this purpose. The total number of ova counted from the thick smear was multiplied by 24 to get the number of ova per gram of stool specimen. This count was used to further classify the infections into light, moderate and heavy infections.

Concentration technique

To prepare the sample, it was mixed with 10% formal-saline in a test tube and then filtered through a 350-450 μm mesh. 7ml of the filtrate was then poured into a 15ml centrifuge tube, and 3ml of diethyl ether was added to the test tube. The mixture was carefully mixed and then centrifuged at 1500 rpm for 4 minutes, resulting in three layers. The top layer (diethyl ether-organic debris mixture) and the supernatant were removed, and a drop of sediment was taken from the remaining layers. This sediment was then placed on a glass slide, covered with a cover slide, and examined under a low-power microscope.

Quality assurance

The laboratory process began with a quality check of the chemicals and materials. The Kato-Katz and concentration techniques were then applied by experienced laboratory technologists for diagnosis.

Data management and analysis

To summarize the data, descriptive statistics such as frequency and percentages were used. The Kato-Katz thick smear preparation was used to count the total number of ova, which were then multiplied by 24 to obtain the number of ova per gram of the stool specimen. This helps in estimating the burden of worm infection and determining the severity of the disease.

Results

Prevalence of intestinal schistosomiasis infection

A total of 597 school-aged children participated in the study. Of these, 308 (51.6%) were female and 289 (48.4%) were male. The median age of the study participant was 8. Out of the 597 children who participated in the study, 312 of them were found to be infected with *S. mansoni*, which accounts for 52.3% (95% CI: 48.4.4-56.4) (Table 1).

Co-infection of *S. mansoni* and other intestinal parasites

The prevalence of co-infection of S. mansoni with other intestinal parasite was 5.1% (30/597). There was co-infection of S. mansoni with all the parasites identified in this study. The study also documented the prevalence of triple infection, specifically *S. mansoni* with *A. lumbricoides* and *Taenia*, as well as *S. mansoni* with *A. lumbricoides* and *H. nana*. The prevalence of single infection of *H. nana* was 1.3% (8/597), *A. lumbricoides*

was 0.7% (4/597), *Taenia* species was 0.5% (3/597) and *G. lamblia* was 0.2% (1/597) (Table 1).

Table 1. Intestinal *Schistosoma* and other intestinal parasites co-infection prevalence among school-age children (n = 597) in Shelle Mela, south Ethiopia (January to July 2023)

Parasites species	Number of cases	Percentage
S. mansoni	312	52.3
H. nana	8	1.3
A. lumbricoides	4	0.7
G. lamblia	1	0.2
Taenia species	3	0.5
S. mansoni + *A. lumbricoides*	9	1.5
S. mansoni + *H. nana*	8	1.3
S. mansoni + *Taenia* species	6	1.0
S. mansoni + *Hookworm*	1	0.2
S. mansoni + *A. lumbricoides* + *H. nana*	1	0.2
S. mansoni + *A. lumbricoides* + *Taenia* species	2	0.3
S. mansoni + *E. histolytica*	1	0.2
A. lumbricoides + *E. histolytica*	1	0.2
S. mansoni + *G. lamblia*	1	0.2
Total	358	60.1

Prevalence of other intestinal parasites infection

Out of 597 study participants, 48 (8%) were positive for other intestinal parasites. Among helminths, the *H. nana* and *A. lumbricoides* were relatively the common species. Prevalence of infections due to *H. nana* was 2.8 % (17/597) and *A. lumbricoides* was 2.8% 17/597) was relatively higher than that of the rest. Prevalence of Hookworm infection was found to be lowest (0.2%). Among protozoans, *G. lamblia* and *E. histolytica* infection were rarely identified (Table 2).

Table 2: Prevalence of other intestinal parasites co-infections among school age children in Shelle Mela, south Ethiopia (January to July 2023).

Parasite species identified	Number of positives	Prevalence
H. nana	17	2.8
A. lumbricoides	17	2.8
Taenia species	9	1.5
Hookworm	1	0.2
E. histolytica	2	0.3
G. lamblia	2	0.3

Parasite intensity

Among school-age children, the mean egg count of parasites was 182.1 eggs per gram. The intensity of *S. mansoni* infection ranged from light to heavy, with light (55%), moderate (30.3%), and heavy (14.7%). The mean number of eggs per gram of stool samples was 725.02 among heavily infected individuals, 195.8 in moderately infected ones, and 44.4 in lightly infected children. On the other hand, the parasite intensity of *H. nana*, *A. lumbricoides, Taenia* species, and Hookworm was moderate.

Discussion

The study aimed to assess the prevalence and burden of *S. mansoni* infection among school-aged children in Shelle Mela, located in south Ethiopia. The results of the study indicate that 52.3% of school-aged children were infected with *S. mansoni*. The infection severity varied from mild to severe, with 55% of the cases classified as mild, 30.3% as moderate, and 14.7% as severe, based on the WHO guidelines.

The study reveals a high infection rate of S. mansoni among school-age children, consistent with other studies in different parts of the country [10–12]. For instance, a 50% infection rate was found in Abbey and Didessa Valleys, Western Ethiopia [13]. Different prevalence rates have been reported across the country, with some areas showing higher prevalence than others [11,12]. Despite this variation, *Schistosoma* infection remains a significant public health concern in the country. The participants in the study have a habit of swimming and washing their clothes on river shores, increasing the risk of *S. mansoni* infection. Therefore, it is vital to maintain a clean environment to minimize water contamination and improve access to clean water.

This study revealed that the overall prevalence rate of intestinal parasites, including co-infections and triple infections, was 8%. The identified soil-transmitted helminths in this area included *H. nana*, *A. lumbricoides*, protozoa such as *G. lamblia*, and *Taenia* species, as documented in several studies across different regions [8]. The prevalence rate of co-infection of *S. mansoni* with other intestinal parasites, including *Ascaris, H. nana, Taenia* species, Hookworm, *E. histolytica*, and *G. lamblia*, was 7.8%. Moreover, this study identified a triple infection of *S. mansoni* with *Ascaris*, *H. nana*, and *Taenia* species. The presence of multiple co-infections of diseases may be linked to poor hygiene conditions [15], indicating a lack of access to clean water and unhygienic conditions as well as contact with contaminated water.

In this study, the severity of infection caused by *S. mansoni* was determined by measuring the number of eggs present in a gram of stool. Light infections were observed in 55.0% of the cases, moderate infections in 30.3%, and heavy infections in 14.7%. These findings are consistent with a previous study conducted in different parts of Ethiopia [16]. The majority (85.5%) of positive cases had mild to moderate infections, possibly due to the initial number of infecting cercariae. These infectious stages are usually found in the lake shore and can enter the bodies of children in small numbers

during brief water contact. *Schistosoma* infection results in premunition, where the initial infection triggers the body to prevent another similar parasite from entering, regardless of its burden [17]. In the study area, there was no deworming program for *Schistosoma* infection, so most children may live with the infection chronically with low to moderate burdens.

The study did not investigate the identification of intermediate hosts and relied on a cross-sectional study design, which may have either overestimated or underestimated the prevalence. Additionally, methodological biases associated with schistosomiasis may have led to an underestimation of the prevalence of other species. However, the procedures were standardized and conducted by experienced laboratory technicians. The study provided valid results to help guide the control programme in reducing the disease burden.

Conclusions

The study identified that more than half of school-aged children were infected with *S. mansoni*, with varying levels of infection severity. Therefore, a school-based deworming programme against *S. mansoni* could be recommended to reduce the disease burden in children.

Acknowledgments

We acknowledge the parents/guardians for consenting to their children's participation in the study and the laboratory technologists for their support.

Author contributions

Conceived the study: YA, FM, TW

Sample collection: YA

Involved in the laboratory work: YA

Analyzed the data: YA, FM

Supervise the laboratory work: DW, FM

Drafted the manuscript: FM

Critically commented on the manuscript: FM, TW, DW

Funding

FM received support from the Norwegian Programme for Capacity Development in Higher Education and Research for Development (QZA-21/0162). The funder had no involvement in the study design, data collection, interpretation, or manuscript write-up.

Competing interests

All authors have declared that they do not have any competing interests.

Ethical approval

The Arba Minch University Institutional Review Board (IRB/1254/2022) approved the study protocol. We also obtained informed written consent and assent from all participants and their parents/guardians. All positive cases were treated in accordance with the national treatment guidelines.

References

1. World Health Organization. WHO GUIDELINE on control and elimination of human schistosomiasis. 2022.

2. Aula OP, McManus DP, Jones MK, Gordon CA. Schistosomiasis with a focus on Africa. Trop Med Infect Dis. 2021; 6: 109.

3. Ajibola O, Gulumbe B, Eze A, Obishakin E. Tools for detection of Schistosomiasis in resource limited settings. Med Sci. 2018; 6: 39.

4. Gryseels B, Polman K, Clerinx J, Kestens L. Human schistosomiasis. Lancet. 2006; 368: 1106–1118.

5. Deol AK, Fleming FM, Calvo-Urbano B, Walker M, Bucumi V, Gnandou I, et al. Schistosomiasis — Assessing progress toward the 2020 and 2025 Global Goals. N Engl J Med. 2019;381: 2519–2528.

6. Kamel B, Laidemitt MR, Lu L, Babbitt C, Weinbaum OL, Mkoji GM, et al. Detecting and identifying schistosoma infections in snails and aquatic habitats: A systematic review. PLoS Negl Trop Dis. 2021;15: e0009175.

7. Negussu N, Mengistu B, Kebede B, Deribe K, Ejigu E, Tadesse G, et al. Ethiopia schistosomiasis and soil-transmitted helminthes control programme: Progress and prospects. Ethiop Med J. 2017;55: 75–80.

8. Leta GT, Mekete K, Wuletaw Y, Gebretsadik A, Sime H, Mekasha S, et al. National mapping of soil-transmitted helminth and schistosome infections in Ethiopia. Parasites and Vectors. 2020;13: 437

9. Olkeba BK, Boets P, Mereta ST, Mandefro B, Debesa G, Ahmednur M, et al. Malacological and Parasitological Surveys on Ethiopian Rift Valley Lakes: Implications for Control and Elimination of Snail-Borne Diseases. Int J Environ Res Public Health. 2022; 19:142.

10. Tefera A, Belay T, Bajiro M. Epidemiology of *Schistosoma mansoni* infection and associated risk factors among school children attending primary schools nearby rivers in Jimma town, an urban setting, Southwest Ethiopia. PLoS One. 2020;15: e0228007.

11. Jejaw A, Zemene E, Alemu Y, Mengistie Z. High prevalence of *Schistosoma mansoni* and other intestinal parasites among elementary school children in Southwest Ethiopia: A cross-sectional study. BMC Public Health. 2015;15: 600

12. Tadege B, Shimelis T. Infections with *Schistosoma mansoni* and geohelminths among school children dwelling along the shore of the Lake Hawassa, southern Ethiopia. PLoS One. 2017;12: e0181547

13. Assefa A, Erko B, Gundersen SG, Medhin G, Berhe N. Current status of *Schistosoma mansoni* infection among previously treated rural communities in the Abbey and Didessa Valleys, Western Ethiopia: Implications for sustainable control. PLoS One. 2021;16: e0247312.

14. Zerdo Z, Bastiaens H, Anthierens S, Massebo F, Masne M, Biresaw G, et al. Prevalence, intensity and endemicity of intestinal schistosomiasis and soil-transmitted helminthiasis and its associated factors among school-aged children in Southern Ethiopia. Sci Rep. 2022;12: 4586

15. Schmidlin T, Hürlimann E, Silué KD, Yapi RB, Houngbedji C, Kouadio BA, et al. Effects of Hygiene and Defecation Behavior on Helminths and Intestinal Protozoa Infections in Taabo, Côte d'Ivoire. PLoS One. 2013;8: e65722

16. Zeleke AJ, Addisu A, Tegegne Y. Prevalence , Intensity , and Associated Factors of Schistosoma mansoni among School Children in Northwest Ethiopia. J Parasitol Res 2020; 2020.

17. Perera DJ, Koger-pease C, Paulini K, Daoudi M, Ndao M. Beyond schistosomiasis:

unraveling co-infections and Altered Immunity. Clin Microbiol Rev. 2024; 37:e0009823
