# Supplementary material for: High prevalence of intestinal schistosomiasis in school-age children in the villages adjacent to Lake Chamo in the southern Rift Valley of Ethiopia
Source: PLoS Negl Trop Dis. 2024 Oct 29;18(10):e0012613. doi: 10.1371/journal.pntd.0012613 (PMC11548786; doi:10.1371/journal.pntd.0012613)
Supplement: Attachment — Submitted filename: Point by point response.docx [file pntd.0012613.s002.docx]

Author Response to Reviewers Comments

We thank the reviewers for their helpful comments and dedication to improving our manuscript.

| **Reviewer’s comments** | **Author response** | **Edited section** |
| --- | --- | --- |
| -Are the objectives of the study clearly articulated with a clear testable hypothesis stated?</br>  -Is the study design appropriate to address the stated objectives?</br>  -Is the population clearly described and appropriate for the hypothesis being tested?</br>  -Is the sample size sufficient to ensure adequate power to address the hypothesis being tested?</br>  -Were correct statistical analysis used to support conclusions?</br>  -Are there concerns about ethical or regulatory requirements being met?</br></br>  Reviewer #1: Yes, although the authors could describe a little more the parameters for the villages selected. They state households within villages randomly selected, which is fine, but not why certain villages surrounding the lake were selected. What was the criteria for deciding if the village was included? was there a maximum distance? Also, some more description of the study area itself, and why it hasn't been included previously in the PZQ program. | The authors have acknowledged the comments and suggestions. | The study area description now includes information on the selection of villages (Lines 106-110).  Apart from the soil-transmitted helminths deworming program, the study area has not been included in the praziquantel MDA due to insufficient evidence on the prevalence and intensity of shistosomiasis. The findings of the current study support the implementation of essential preventive measures for those at higher risk of infection. This data could assist in efforts to prevent the disease at healthcare facilities and within various governmental and non-governmental organizations (Lines 84-89) |
| Reviewer #3: -Are the objectives of the study clearly articulated with a clear testable hypothesis stated?  Neither the objectives nor study hypothesis were stated. | We appreciate the feedbacks and have made efforts to address them. | We believe that the following is the objective of the study: **‘’**Therefore, the current study aimed to determine the prevalence and intensity of intestinal schistosomiasis among school-age children living in selected villages near Lake Chamo in the southern Rift Valley lakes of Ethiopia’’ (Lines 92-94)  We also hypothesised that ‘’the school-age children are more likely to make swimming a regular activity and, therefore, may be at a higher risk of infection than other groups’’ (lines 80-82). Therefore, the study targeting this age group allows for a better understanding of the disease's prevalence and suggests possible interventions (lines 81-83) |
| Is the study design appropriate to address the stated objectives?  Although a cross-sectional study design is suitable for assessing prevalence, it's challenging to evaluate alignment with the study's objectives without knowing them.  -Is the population clearly described and appropriate for the hypothesis being tested?  SAC 5-15 years was cited as study population, but no hypothesis was stated.  -Is the sample size sufficient to ensure adequate power to address the hypothesis being tested?  Hard to tell. The estimated sample size was 597 SAC, the total population of the study area is unknown. Again, no hypothesis was cited.  -Were correct statistical analysis used to support conclusions?  Inferential and descriptive statistics were used, but the conclusions were somehow vague.  -Are there concerns about ethical or regulatory requirements being met?  I would assume ethical concerns were addressed since the Arba Minch University Institutional Review Board approved the study protocol and informed written consent and assent was obtained as needed. Also, positive cases were treated in accordance with the national treatment guidelines. | The authors are grateful for the comments and suggestions. | We made an effort to state the objective of the study and hypothesis clear (Lines 92-94). The hypothesis is also indicated (lines 80-81).  The sample size calculation was done following the standard formula for single population proportion and the authors believed that the sample size is sufficient to address the proposed objective. The random selection of house households and inclusion of all school age children in the selected households ensure representativeness and avoid selection bias (we included this in the last paragraph of the discussion section).  Additional information about sampling households and individuals was also included (Lines124-127). |
| Does the analysis presented match the analysis plan?</br>  -Are the results clearly and completely presented?</br>  -Are the figures (Tables, Images) of sufficient quality for clarity?</br></br>  Reviewer #1: Yes, apart from the following: 1) the figure. Please describe what is being presented in the figure either in text or expand on the figure description.  2) It would be good to present the intensity as % prevalence of whole sample. ie ~28% of SAC in Shele Mela Kebele have light intensity infection X% moderate etc. This is because the WHO guidelines for elimination as a public health problem are tied to data presented in this manner | We appreciate the comments and suggestions | We are unclear about which figure the reviewer is referring to. The legends would explain it well if it were the study area map. Regarding the intensity, we presented the light, moderate and heavy infection levels along with the corresponding prevalence and 95% confidence intervals. (Lines 222-225) |
| Reviewer #2: All prevalence estimates need to be accompanied by their 95% confidence intervals.  The results of the questionnaire also need to be presented. | The comments are well accepted. | We have calculated the 95% confidence interval for all prevalence data (see all tables and results presented)  The questionnaire was designed to gather the age and gender of the study participants. We focused on determining the prevalence and intensity of infection to provide evidence for action. More data on risk factors and related would have been beneficial. |
| Reviewer #3: -Does the analysis presented match the analysis plan?  No- The paper didn't demonstrate an analysis plan.  -Are the results clearly and completely presented?  Despite findings being presented, it's hard to ascertain if they were complete as the study objectives were not stated. Also, it's unclear why urinary schistosomiasis wasn't investigated. It would've been logical to cite previous studies revealing the non-endemicity of urinary schistosomiasis if that was the case in the study area. On the contrary, data from the WHO/ESPEN portal reveals Arba Minch district (the area under investigation) is endemic to urogenital schistosomiasis.  Kato-Katz and formal ether concentration techniques were both used for stool analysis.  It’s unclear if:  -    the outcomes were different by technique,  -    one technique was used as first line screening and the other for confirmatory diagnosis.  It is necessary to state:  -    why both techniques were used  -    if prevalence/intensity varied by technique  -    how prevalence and intensity were established using both diagnostic methods.  -Are the figures (Tables, Images) of sufficient quality for clarity?  Yes- Tables and narratives are clear. However, the results on prevalence and intensity were not disaggregated by gender or school enrolment/attendance. Also, it would've been excellent to appraise the knowledge of survey respondents on disease transmission and prevention. Furthermore, data wasn't disaggregated by the five sub villages in the Shelle Mela Kebele area. | We appreciate the feedback and suggestions.  We appreciate the comments regarding urinary schistosomiasis, which is an important point.  These comments are of utmost importance. Thank you for highlighting these points. | We utilized only descriptive statistics to estimate the prevalence and intensity of the infection, in line with the study objective.  Our study plan did not take into account urinary schistosomiasis. It would be beneficial to include it, as it may yield evidence for this disease control programme.  Yes, we utilized both techniques for stool sample analysis. We used this technique if parasites were missed by the Kato-Katz technique for treating cases, not for reporting results, as our primary focus was intestinal schistosomiasis (Line 177-179). The Kato-Katz result was reported here, as our primary focus was intestinal Schistosomiasis.  However, we value the lesson that providing data on different techniques can help recommend better diagnostic methods for the public.  The study was community-based (Lines 113-115) but focused on school-age children rather than specifically school-enrolled children. The children are believed to be mainly from the same school, as Kebele has one primary school. The village was divided into smaller sub-village to ensure the sample of households selected from the health post registration was representative, but this was not done for the purpose of analyzing the data based on the sub-village (Lines 120-125) |
| -Are the conclusions supported by the data presented?</br>  -Are the limitations of analysis clearly described?</br>  -Do the authors discuss how these data can be helpful to advance our understanding of the topic under study?</br>  -Is public health relevance addressed?</br></br>  Reviewer #1: Yes, though please provide the recommendation of school-based deworming in the area with a brief justification in the discussion, not just the conclusion | The authors appreciate these comments. | We provided a short recommendation of deworming, explanations, and future sustainability prospects for deworming (Lines 237-242) |
| Reviewer #3: -Are the conclusions supported by the data presented?  Partially- It was concluded that 52.3% of SAC were infected with intestinal schistosomiasis, but the absence of S. haematobium in the study makes it challenging to conclude if S. mansoni is the predominant species in the study area.  Also, it would be useful to confirm if STH deworming is required or not, given that Kato-Katz and formol ether techniques were used for stool analysis.  -Are the limitations of analysis clearly described?  No- Study limitations were not mentioned.  -Do the authors discuss how these data can be helpful to advance our understanding of the topic under study?  Partially - Study falls short of investigating transmission factors like access to water sanitation and hygiene, including behavioural determinants of transmission amongst participants.  -Is public health relevance addressed?  Partially - Beyond MDA being recommended, the need for health education initiatives was not cited. | We acknowledge the comments and suggestions. | As the reviewer noted, our conclusion was based on the finding of intestinal schistosomiasis, which was our objective. No comparison was made with *S. haematobium*. However, we acknowledge the inclusion of *S. haematobium* in future studies.  School based STH deworming programme is already in place in the study area (Line 84-85)  In the last paragraph of our discussion, we presented certain study limitations, which we have further expanded on in the revised version (Lines 270-283)  We incorporated additional elements beyond MDA to further guarantee the deworming program's sustainability (Lines 288-290) |
| Reviewer #1: As above, please provide prevalence of intensity class using total sampled as denominator as well as using total infected as denominator which is already presented | Thank you for these comments. | We provided data on the prevalence of intensity among the positive cases; however, we are uncertain about the specific information obtained by providing the prevalence of intensity among the total cases screened. |
| Reviewer #3: Hyperlinks to sample consent forms, IRB requirements and questionnaires could better enhance the appraisal of reviewers. | Thank you for this suggestion. | We are uncertain about the specific benefit of hyperlinking the consent forms and questionnaires. We will upload the ethical approval letter when we submit the revised version. |
| Reviewer #1: Authors have presented an important and well performed survey of S. mansonii (SCH) in a seemingly underserved area of Ethiopia. The alarmingly high prevalence and intensity of SCH within the targeted communities will hopefully provide Government with sufficient evidence to roll out preventative chemotherapy to avoid further morbidity and mortality. The authors are commended on their effort. | Thank you for this feedback, We appreciate | We provide the data to the relevant authorities in the hope that they will take appropriate action. |
| Reviewer #1: A few minor comments, further to those above:  - Please provide a thorough review language used  - Please ensure references are used where appropriate, for example - methods section states health center registration records were checked. Convention states that a reference should be provided here, even if it is a government data set.  - Methods suggest only households with SAC were included in study. How were households with SAC identified. Was a community register used, then random households selected?  - Could authors provide the timing from sample collection to slide reading? This is important for results relating to hookworm - although identifying hookworm is not the primary objective of the study, would be useful nonetheless  - The calculation for the epg in the methods is repeated (ie mentioning multiplication by 24) this need only be in the article once  - Participant selection suggests samples given only upon consent, please expand a little here. Was informed consent involving information on the study objectives etc given prior to offering participation?  - IRB approval has been obtained for this study, but a statement of the IRB process/approval should be provided in the methods section.  - Please review the discussion section for some repetition | The authors acknowledge the reviewer for providing this feedback. | - We have thoroughly reviewed the documents for language and other editorial issues.  -We have checked the references  -The health Centre data was just a record on the log book  -The details on how the households and SAC were selected were included in the method section (Lines 122-125).  -  We obtained the approval letter before we start the study  The discussion section is reviewed carefully. |
| There are no line numbers, making it very difficult to provide detailed comments.  There are some typos and grammatical errors that will need to be corrected. Kindly go through the entire text and address them. See the attached for further comments.  The discussion and recommendation could be expanded to consider the treatment of out-of-school children and other community members. Other control options could also be discussed.  The data on the socio-demographic information (other than age and gender) was not presented. The questionnaire should be added as an appendix to the study, and the rest of the data presented.  Please italicize S. mansoni and other scientific names throughout the text. | Thank you for the comments | Line numbers provided.  The authors have reviewed the entire text to address typos and grammar mistakes. The study involved school-age children within the community, and our recommendations and discussions are beyond this age group. The scientific names are italicized. |
| A better word may be "infected" | Thank you for suggesting a better word | We have substituted “contamination” with “infected” |
| Title: The second part of the title is not relevant. The area has not received any treatment before. Thus, the high prevalence should automatically indicate the need for a control approach. It is not a question for debate. This is not relevant. The area has not received any treatment before. Thus, the high prevalence should automatically indicate the need for a control approach. It is not a question for debate. | Thank you for the suggestion | We removed the second part. |
| Add this as an appendix | Thank you for the suggestion | Unfortunately, we didn’t see particular benefit from adding the questionnaire other than increasing the volume. |
| “remaining portion” for “the rest portion” | Thank you for the suggestions | We have made some modification |
| Move “The total number of ova counted from the thick smear was multiplied by 24 to get the number of ova per gram of stool specimen. This count was used to further classify the infections into light, moderate and heavy infections.” to data analysis section | Thank you for the suggestion | It is moved to the data analysis section (Lines 188-192) |
| Provide 95% CIs for all prevalence estimates for tables 1 and 2. | Thank you for the suggestion. | We have done it (see all the tables) |
| Reviewer #2: This is a straightforward parasitological study assessing |  |  |
| The notion “initial number of infecting cercariae might be the reason for mild to moderate infection” is wrong. Infection and egg counts increase over time. | Thank you for comments | We believe that the severity of infection and the parasite burden depend on the original number of infecting larvae. In other words, all the adult parasites originated from those larvae, and their numbers don't increase. These parasites are the only source of the released eggs. While egg production may increase shortly after infection, it tends to remain constant as the infection becomes chronic. |
| Rephrase the sentence “These infectious stages are usually found in the lake shore and can enter the bodies of children in small numbers during brief water contact” | Thank you so much for this suggestion | Cercariae are found where the intermediate host (snails) is present and could potentially infect children in small numbers as they enter and exit the lake, typically involving brief water contact (Lines 264-266) |
| What is meant by Premunition? | Thank you for the quarries. Hope we explained it properly | “Premunition, in this context, refers to the immunity against additional *Schistosoma mansoni* infection in individuals who have already tested positive for the infection. The existing infection, regardless of its severity, triggers strong immunity against new infections. However, re-infection may occur if the infected individual is treated and becomes free from the worm." |
| Explain how “cross-sectional study design, which may have either overestimated or underestimated the prevalence.” | Thank you for requesting an explanation. | The study also used a cross-sectional design, which may lead to over- or underestimation of prevalence as it captures only the current situation and may need to accurately reflect changes over time (Lines 272-274). We considered this as a limitation for this study |
| Explain this statement “methodological biases associated with schistosomiasis may have led to an underestimation of the prevalence of other species” | The authors appreciated the comments and made the indicated modifications | The study did not report the results of the concentration technique to compare with the Kato-Katz method (Lines 273-275). This method might not be the best option for other intestinal parasites, although it is the right choice for abdominal schistosomiasis. Therefore, the results may not accurately reflect the prevalence of other intestinal parasitic infections. |
| Write “is recommended” instead of “could be recommended” | Thank you for the comment | We have incorporated the comment |
